# Evaluation of peripapillary vascular flow in patients with Thyroid-Associated Ophthalmopathy (TAO) by OCT Angiography

**DOI:** 10.1007/s00417-022-05551-7

**Published:** 2022-02-12

**Authors:** Chiara Del Noce, Matilde Roda, Nicola Valsecchi, Sofia Guandalini, Natalie Di Geronimo, Costantino Schiavi, Carlo Enrico Traverso, Aldo Vagge

**Affiliations:** 1 Department of Neuroscience, Rehabilitation, Ophthalmology, Genetics, Maternal and Child Health (DINOGMI), University Eye Clinic of Genoa, IRCCS Ospedale Policlinico S. Martino, Genova, Italy; 2 Ophthalmology Unit, Department of Experimental, Diagnostic and Specialty Medicine (DIMES), Alma Mater Studiorum University of Bologna and S.Orsola-Malpighi Teaching Hospital, Bologna, Italy

**Keywords:** Thyroid-associated ophthalmopathy, Graves’ disease, OCTA, Peripapillary vascular flow

## Abstract

**Purpose:**

To evaluate changes in peripapillary vascular blood flow indices (PVBFI) in patients with thyroid-associated ophthalmopathy (TAO) using OCT angiography (OCTA) technology.

**Methods:**

Patients with TAO and control subjects matched for age and sex were included in the study. Eye examination, Clinical Activity Score (CAS) evaluation and OCTA scan analysis (Topcon ImageNet 6; DRI OCT Triton, Topcon Corporation) were performed. In particular, PVBFI of the superficial capillary plexus (SCP), deep capillary plexus (DCP), outer retina (OR) and choriocapillaris (CC) layers were obtained by OCTA and extracted from 8-bit greyscale OCT images using the ImageJ software package.

**Results:**

Twenty-six patients with TAO (19 females, mean age 54.7 ± 5.2 and 7 males, mean age 51.4 ± 16.3) were compared with 26 healthy subjects (15 females, mean age 48.2 ± 14.1 and 11 males, mean age 53.1 ± 15.2). Both DCP-PVBF and CC-PVBFI were significantly reduced in TAO patients compared to control eyes (28.6 ± 2.1 vs. 29.7 ± 0.93, *p* = 0.002; 46.5 ± 1.72 vs. 47.2 ± 1.2, *p* = 0.019 respectively); on the other hand, no statistically significant differences were found in SCP-PVBFI and OR-PVBFI in TAO patients compared to healthy subjects (*p* > 0.05). Also, CC-PVBFI was associated with elevated values of CAS (*p* = 0.018) and ROC curve showed that patients with elevated CC-PVBFI were correlated with active TAO (CAS > 3) (*p* = 0.012).

**Conclusions:**

TAO disease may be associated with changes in DCP-PVBFI and CC-PVBFI; also, CC-PVBFI seems to correlate with disease activity.


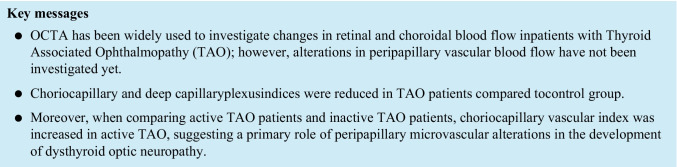


## Introduction

Thyroid-associated ophthalmopathy (TAO) is a systemic autoimmune disease with a broad spectrum of signs and symptoms [1]. Ocular and thyroid symptoms often occur simultaneously; however, sometimes ophthalmopathy precedes or follows the onset of hyperthyroidism [2]. Clinical assessment has been made traditionally by the “NO SPECS” classification (Table 1), in order to easily classify the severity of the disease. Nevertheless, this classification was not reliable in defining the progression of the disease; therefore, Mouritis et al.1989 introduced the Clinical Active Score (CAS) (Table 2), which allows to distinguish the disease in active and inactive forms by considering seven different parameters that suggest grades of activity of the disease [3].

**Table 1:** NO SPECS classification (VA = Visual Acuity)

Score	Finding
0	**N**o signs and symptoms
1	**O**nly signs
2	**S**oft tissue involvement with symptoms and signs
3	**P**roptosis (≥ 20 mm)
4	**E**xtraocular muscle involvement
5	**C**orneal involvement
6	**S**ight loss (VA ≤ 0.67)

**Table 2:** Values of peripapillary vascular flow indices in superficial capillary plexus, deep capillary plexus, outer retina and choriocapillaris layers between patients with TAO and control group. SD = Standard Deviation

	TAO group	Control group	*p* value
Superficial capillary plexus, mean (SD)	39.57 (3.09)	40.4812 (1.6149)	*p* = 0.082
Deep capillary plexus, mean (SD)	28.60 (2.10)	29.7213 (0.93685)	*p* = 0.002
Outer retina plexus, mean (SD)	37.99 (2.94)	37.8758 (1.35873)	*p* = 0.798
Choriocapillary plexus, mean (SD)	46.52 (1.72)	47.221 (1.20444)	*p* = 0.019

Periorbital tissues are mostly affected, and the most common findings are interstitial edema, orbital fat hyperplasia and swollen of extraocular muscles; other common symptoms are upper eyelid retraction, diplopia and proptosis, which are usually bilateral and often asymmetric [4].

Hemodynamic changes in these patients have been studied in several different ways, such as Heidelberg retina flowmetry, ocular blood flow tomography, oculo-dynamometry and color Doppler imaging (CDI) [5]. CDI is a noninvasive approach, which has been widely used to assess orbital vessels blood flow; nevertheless, there are several influencing factors in the measuring process, such as the pressure applied on the eyeball, eye movements and sampling volumes.

Optical coherence tomography (OCT) is a rapid and noninvasive technique that allows detailed structural visualization of retina and choroid; moreover, OCT angiography (OCTA) studies retinal and choroidal vascularization by producing three-dimensional microcirculation vascular maps without the use of dye [6]. Through the en face presentation of the volumetric angiogram, it is possible to measure areas of interest and to quantity density of vessels in the macula or blood flow in specific areas of the retina [7].

Retinal and choroidal blood flow of patients with Graves’ ophthalmopathy has been largely investigated in previous studies in order to identify changes in foveal and parafoveal microvascular density and retinal vessel caliber [8], choroidal thickness and choroidal vascular blood flow, retinal nerve fiber layer (RNFL) thickness and choroidal and macular thickness [9, 10]. To the best of our knowledge, peripapillary microvascular flow was never investigated in patients with TAO, although it could be involved in the development of the optic nerve damage. Therefore, the aim of this study was to evaluate changes in peripapillary vascular blood flow using OCTA, in order to identify differences between control and TAO patients and between active TAO and inactive TAO patients, in order to clarify the role of peripapillary blood flow in the pathogenesis of the disease.

## Material and Methods

The study was conducted at the University Eye Clinic of Genoa, Department of Neuroscience, Rehabilitation, Ophthalmology, Genetics, Maternal and Child Health (DINOGMI), IRCCS Ospedale Policlinico S. Martino, Genova, Italy. In this study, 26 participants with the diagnosis of Graves–Basedow disease and thyroid-associated ophthalmopathy (TAO) were recruited; the same number of subjects was enrolled to assemble the control healthy group. All participants gave their informed consent, and the study was conducted in accordance with the tenets of the Helsinki Declaration. Including criteria were age ≥ 18, Graves’ disease diagnosis, refractive error lower than 3 diopters of spherical equivalent and intra-orbital pressure (IOP) less than 20 mmHg, measured with Goldmann applanation tonometer. Patients with ocular diseases that affected choroidal thickness such as glaucoma, uveitis, retinal and choroidal diseases were excluded, same as for patients who underwent intraocular surgery.

Each subject underwent a comprehensive eye examination including anamnesis, best-corrected visual acuity (BCVA), slit lamp examination of the anterior segment, indirect ophthalmoscopy, Hertel exophthalmometry, OCT and OCTA.

TAO activity was assessed through the CAS score, and patients were divided into non-active TAO (CAS < 3) and active TAO (CAS ≥ 3).

**Fig. 1:**
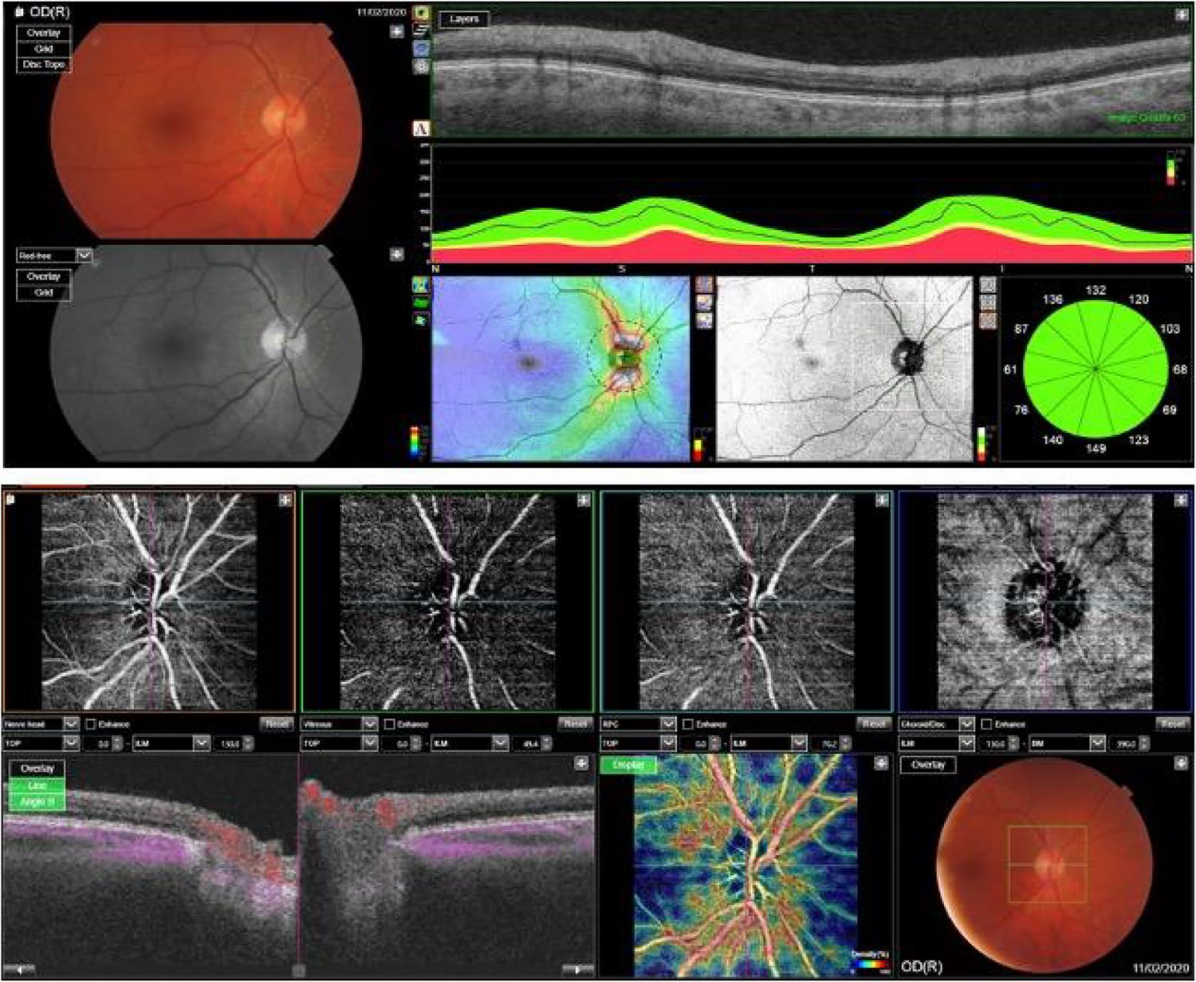
OCT Topcon ImageNet 6 (DRI OCT Triton, Topcon Corporation) was used to obtain OCT swept- source angiography scans from an area of 4.5 × 4.5 mm^2^ centered on the optic nerve. Four layers were considered: superficial capillary plexus (SCP), deep capillary plexus (DCP), outer retina (OR) and choriocapillaris (CC). ImageJ software was used to calculate the peripapillary vascular index. Pictures were converted from black and white into binary images; afterward, colors channel was adjusted through “color threshold” in order to visualize vascular structures. Peripapillary region was obtained by drawing two ellipses around it (270X230 nm and 60X60 nm), visualizing the Vascular Index of the interested area

OCT swept-source angiography scans were obtained using OCT Topcon ImageNet 6 (DRI OCT Triton, Topcon Corporation) from an area of 4.5 × 4.5 mm^2^ centered on the optic nerve. For each patient were considered images of four layers: superficial capillary plexus (SCP), deep capillary plexus (DCP), outer retina (OR) and choriocapillaris (CC). Each image was used to calculate, through the ImageJ software, the peripapillary vascular index. Pictures were converted from black and white into binary images; afterward, colors channel was adjusted through “color threshold” in order to visualize vascular structures. Eventually, it was possible to obtain peripapillary region by drawing two ellipses around it (270 × 230 nm and 60 × 60 nm), visualizing the Vascular Index of the interested area. See Fig.1.

For statistical analysis, normality was tested with Shapiro–Wilk test and parametric test were used. T-Test was used to compare vascular indices between TAO patients and control groups and to test vascular indices between men and women. Pearson’s correlation coefficient test was run to evaluate correlations between Hertel exophthalmometry, IOP, CAS score, visual acuity and vascular indices.

One-way ANOVA was run to evaluate the difference between active TAO and inactive TAO patients for vascular indices. Receiving operating characteristic (ROC) curves were obtained in order to evaluate cutoff values.

*P* values < 0.05 were considered statistically significant.

Statistical analysis was performed using IBM Statistical Package for Social Sciences version 26.

## Results

Twenty-six patients with TAO (mean age 53.8 ± 15.2) divided in 19 females (mean age 54.7 ± 5.2) and 7 males (mean age 51.4 ± 16.3) were compared with 26 healthy subjects divided in 15 females (mean age 48.2 ± 14.1) and 11 males (mean age 53.1 ± 15.2). Median time at diagnosis was 23 months. 24 patients with TAO were taking a daily intake of 15 mg methimazole, whereas 2 patients were taking 30 mg of methimazole daily. Except for 8 patients who were taken drugs for systemic hypertension, no other relevant concomitant diseases were presented in the population included in the study. At the OCT scan, patients had no alterations in macular morphology, nor in RNFL and ganglion cell complex (GCC) thickness. None of the patients included in the study had compressive optic neuropathy.

Hertel exophtalmometry ranged from 13 to 26 mm (mean 20.51 mm, SD 3.49), whereas mean IOP was 14.26 (SD 2.65); at clinical assessment, mean visual acuity was 0.0 logMar (SD 0.08) and mean CAS was 3.11 (SD 1.36). 8 patients (30.8%) had inactive TAO (CAS < 3), whereas 18 patients (69.2%) had active TAO (CAS ≥ 3).

Values of DCP were significantly reduced in TAO patients compared to control eyes (28.6 ± 2.1 vs. 29.7 ± 9.3, *p* = 0.002, paired t-test); also, values of CC were reduced compared to control group (46.5 ± 1.72 vs. 47.2 ± 1.2, *p* = 0.019). On the other hand, no statistical correlation was encountered in SCP and OR between TAO patients and control group. See Table 2. No statistically significant results were encountered when vascular indices were compared between men and women with TAO. See Table 3.

**Table 3:** Values of peripapillary vascular flow indices in superficial capillary plexus, deep capillary plexus, outer retina and choriocapillaris layers between men and women with TAO. SD = Standard deviation

	Women	Men	p-value
Superficial capillary plexus, mean (SD)	39.862 (3.47)	40.203 (40.2)	0.273
Deep capillary plexus, mean (SD)	28.925 (2.47)	28.714 (1.84)	0.578
Outer retina plexus, mean (SD)	38.351 (3.20)	37.709 (1.66)	0.099
Choriocapillary plexus, mean (SD)	46.698 (46.69)	47.147 (2.33)	0.201

Comparing peripapillary vascular indices, CAS, IOP, Hertel and BCVA, we observed that CC was associated with elevated values of CAS (ρ = 0.462, *p* = 0.018, Pearson’s correlation). Also, a significant correlation was observed between Hertel exophthalmometry values and CAS score in TAO patients (r = 0.80, *p* < 0.001). On the other hand, no statistical correlations were encountered comparing CAS, IOP, BCVA and vascular indices (*p* > 0.05, Pearson’s correlation).

**Fig. 2:**
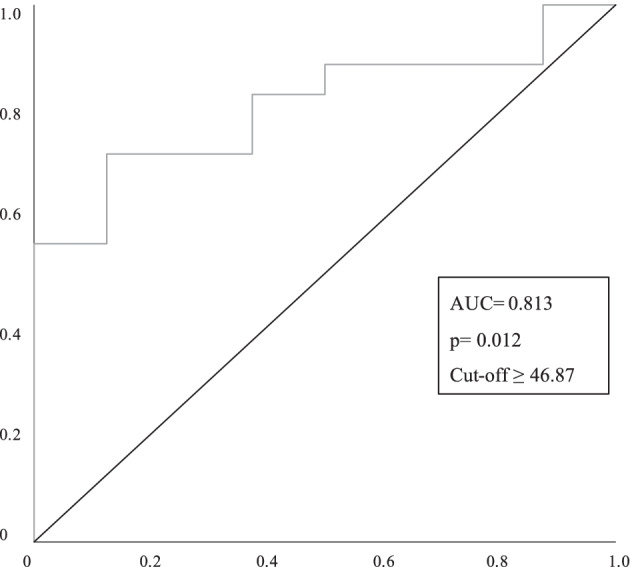
ROC curve analysis was performed to test cutoff value of CC vascular flow index sufficient to discriminate between patients with active and inactive TAO. TAO Patients with ≥ 46.87% of CC peripapillary vascular flow index are more likely to present activity of the disease. The area under the curve (AUC), cutoff values and *p*-values are listed in the panel

Regarding CC plexus, there was a statistically significant difference between active and inactive TAO patients as determined by one-way ANOVA (active TAO mean 47.36, SD = 1.74; inactive TAO patients mean 45.58, SD = 1.21; *p* = 0.016). ROC curve showed that patients with elevated CC vascular indices were correlated with active TAO (CAS > 3), as area under curve (AUC) was 0.813, Youden’s index was 0.597 and cutoff value was 46.87 (*p* = 0.012, ROC analysis). On the other hand, neither SCP, DCP and OR were associated with active TAO. See Fig. 2.

## Discussion

TAO is a common orbital disorder that can lead to dysthyroid optic neuropathy (DON) by compression of the optic nerve by congested tissues and extraocular muscles. The role of vascular blood flow in the pathogenesis of optic neuropathy has been widely investigated with several methods; different studies with Doppler ultrasonography have demonstrated that increase in intra-orbital pressure in TAO patients leads to severe stasis in the superior ophthalmic vein [11–13]; also, other studies observed an increased resistance in ophthalmic artery and in central retinal artery in TAO patients, which decreases after orbital decompression [14, 15]. Some studies with OCTA observed a reduction in macular density in patients with active and inactive TAO [16], other studies observed a greater macular density in inactive TAO patients [17] and other studies showed an increase in macular density in active TAO patients [18]. Also, several studies showed a reduction of fibers in the RNFL layer [19] and recent studies showed that patients with active TAO present with choroidal thickness [10, 17, 20].

The main result of our study is that peripapillary CC is decreased in TAO patients compared to control groups (*p* = 0.019); also, we observed that CC was increased in active TAO patients compared to inactive TAO (*p* = 0.016), suggesting that CC alterations are firstly involved in the active phase of TAO. Decrease in CC density in TAO patients compared to control group could be secondary to the vascular obstruction determined by extraocular muscles and soft tissues; on the other hand, active TAO is characterized by an hyperdynamic cardiovascular state that could explain elevated values of CC in active TAO patients compared to inactive TAO.

Also, we observed that DCP was significantly decreased in TAO patients compared to control group (*p* = 0.002). However, we observed that changes in peripapillary vessels did not interfere with visual acuity and intraocular pressure, suggesting that optic nerve damage has a complex and multifactorial pathophysiology.

To our knowledge, this is the first investigation that studies changes in peripapillary vascularization in TAO patients using OCTA. In accordance with our findings, a recent study by Wu [21] showed that both inner intra-retinal layers around the macula, superficial and deep capillary plexus were lower in TAO patients with DON compared to TAO patients without DON and control group, suggesting the idea that retinal alterations occur prior to changes in visual acuity. Moreover, Ye [18] showed that macular microvascular flow was significantly increased in active TAO patients compared to control group. In line with these findings, our study demonstrates that microvascular alterations also occur in the peripapillary region, suggesting the hypothesis that OCTA could be an useful tool in detecting activity of the disease and in recognizing those patients that are at risk of developing DON.

The main limitation of this study was the small number of patients enrolled; secondary, TAO patients were not divided according to the presence of DON in order to quantify the effect of microvascular alterations in accordance with clinical assessment. Another limitation was that most of the women included in the study were in the perimenopause age. Lucas et al. showed that hot flashes are often accompanied by a reduction in brain blood flow; hence, our results could be altered by the physiological hormonal and hemodynamic changes that occur in pre-menopause age in women [22].

Future studies are needed to confirm our findings with the goal of identifying first alterations in optic nerve using OCTA, in order to prevent DON and irreversible loss of visual acuity.

## Conclusions

CC and DCP were reduced in TAO patients compared to control group. However, when comparing active TAO patients and inactive TAO patients, choriocapillary vascular index was increased in active TAO, suggesting a primary role of peripapillary microvascular alterations in the development of DON.
